# A multi-site, randomized controlled trial of MS INFoRm, a fatigue self-management website for persons with multiple sclerosis: rationale and study protocol

**DOI:** 10.1186/s12883-019-1367-6

**Published:** 2019-06-25

**Authors:** Marcia Finlayson, Nadine Akbar, Karen Turpin, Penny Smyth

**Affiliations:** 1 School of Rehabilitation Therapy, Queen’s University, Louise D. Acton Building,31 George Street, Kingston, Ontario K7L 3N6 Canada; 2 School of Public Health, Department of Public Health Sciences, University of Alberta, Edmonton, Alberta Canada; 3 Division of Neurology, Department of Medicine, University of Alberta, Edmonton, Alberta Canada

**Keywords:** Multiple sclerosis, Fatigue, Self-management, Internet

## Abstract

**Background:**

Fatigue is one of the most common and debilitating symptoms of multiple sclerosis (MS). The majority of approaches for managing MS fatigue typically require participation in a structured, time-limited program with a fixed sequence of topics and activities. MS INFoRm (Multiple Sclerosis: An *In*teractive *F*atigue Management *R*esource) is a self-directed MS fatigue management resource incorporating principles of self-management and adult learning. Positive results from a feasibility pilot study of a USB-delivered version of MS INFoRm led to the current trial and adaptation of MS INFoRm to a website format. The specific aims of the proposed study are to (a) to determine the effectiveness and efficacy of 3-month use of MS INFoRm on fatigue impact (primary outcome) among persons with MS, (b) to determine whether 3-month use of MS INFoRm results in improvement in secondary outcomes of self- efficacy for managing MS fatigue, self-reported cognitive function, participation and autonomy, and depression, and (c) to determine whether any improvements in primary and secondary outcomes are maintained among the MS INFoRm users after 6-months.

**Methods/Design:**

Parallel group, two arm, double-blinded superiority trial with a 1:1 allocation. Two hundred persons with MS will be randomly assigned to either an intervention (MS INFoRm) or usual care control group in which they will be given 3-month access to either the MS INFoRm website (intervention group) or a control webpage containing widely available resources on MS fatigue (control group). Baseline, immediate post-intervention (3-months), and follow-up (6-months post intervention) evaluations will take place on primary (Modified Fatigue Impact Scale) and secondary (Multiple Sclerosis Self-Efficacy Scale, Perceived Deficits Questionnaire, Center for Epidemiologic Studies Depression Scale, and Impact on Participation and Autonomy Questionnaire) measures. Hypothesis testing will involve independent samples t-tests and mixed effects ANOVAs.

**Discussion:**

People with MS may benefit from easily accessible and self-directed fatigue management resources based on self-management and adult learning principles. The proposed study will provide crucial evidence about the potential of MS INFoRm as a self-management tool that can be made widely available to persons with MS as a means to effectively reduce the daily impact of MS fatigue.

**Trial registration:**

ClinicalTrials.gov: NCT03362541. Posting date December 5, 2017.

## Background

People with MS experience a wide range of symptoms, including fatigue [1]. At least two-thirds of people with MS report clinically significant fatigue [2] that negatively impacts employment [3], quality of life [4] and ability to engage in a full range of daily activities [5, 6]. People who have MS describe fatigue as frustrating, overwhelming, and disabling [7]. Presence of MS fatigue has been linked to increased risk of falls [8], comorbidities [9], and use of health care services among people with the disease [10].

MS fatigue has both primary and secondary components, that is, it is both intrinsic to the disease and a function of factors such as deconditioning, poor sleep and nutrition, and stress [6, 11]. As a consequence, management of this symptom can be challenging. To date, treatments have included medications and the rehabilitative approaches of energy management training, cognitive behavioral therapy, and exercise [12, 13]. Across these options, medications are the least effective [14]. Trials of rehabilitative options have uncovered effect sizes in the moderate range [14], with energy management training and cognitive behavioral therapy demonstrating effectiveness for a broader range of people with MS (e.g., levels of disability, age, etc.) [14].

The major limitation of all existing and effective approaches for managing MS fatigue is that they require the individual with MS to participate in a structured, time-limited program that has a fixed sequence of topics and activities. In some cases, participation requires travel to a treatment site [15, 16]. Although internet options exist [13, 17], regular and scheduled participation are still required. Adult learning theory suggests that self-directed approaches to learning and behaviour change may be more effective than structured approaches because they allow adults to maintain their own self-concept; build on life experiences, social roles and motivations; and facilitate immediate application of knowledge in daily life [18]. In addition, literature on self-management suggests that supporting individuals to take control of their chronic conditions leads to better health outcomes [19, 20]. Together, the limitations of existing interventions, knowledge of adult learning theory, and principles of self-management led our team to ask whether a self-directed MS fatigue management intervention would enable people with MS to reduce the impact of this symptom on their daily life. In response, we developed MS INFoRm (Multiple Sclerosis: An *In*teractive *F*atigue Management *R*esource) which incorporates approaches previously identified as being effective for managing MS fatigue [14].

The initial version of MS INFoRm (initially developed in 2012) was an interactive Microsoft PowerPoint Presentation with embedded hyperlinks to worksheets and pdf files, with the entire resource provided via USB key. The contents of MS INFoRM include ways to monitor fatigue, information on how to effectively communicate with others about MS fatigue and when and where to go for help. It also explains several common secondary sources of fatigue (depression, sleep disorders, medications, lack of physical activity, mental exertion) and offers evidence-based energy management strategies that can be used in the context of daily life. We have published pilot findings from the original USB version of MS INFoRm in which 35 individuals with MS who reported mild to moderate fatigue were provided with MS INFoRm to use at home for 3 months, on their own volition [21]. Participants reported actively using MS INFoRm over this period. Three-month use of MS INFoRm was associated with significant reductions in fatigue impact, increased knowledge of MS fatigue, and greater confidence in managing MS fatigue. Our pilot study also included qualitative analysis of semi-structured interview with participants, which revealed that 3-month use of MS INFoRm was associated with shifts in knowledge, expectations, and behaviours with respect to fatigue management. These shifts led to multiple positive outcomes, including increased levels of self-confidence and improved quality of life [22].

Given our promising pilot results, the need for easily accessible interventions that can be used independently to support self-management skill development, we will now conduct a further larger randomized controlled trial of a website version of MS INFoRm. The trial will randomly assign an anticipated 200 participants to one of two conditions: (1) 3-month access to the MS INFoRm intervention website and (2) 3-month access to a “usual care” control website that provides widely available fatigue management resources. The control website was designed to represent the usual care and resources that persons with MS are directed to by their physicians or other health care providers. The specific aims and hypotheses are described below.

### Specific aim 1

Our first aim is to determine the effectiveness and efficacy of 3-month use of MS INFoRm on fatigue impact among persons with MS. Our primary outcome will be measured using the Modified Fatigue Impact Scale (MFIS) [23]. We hypothesize that the MS INFoRm group will show significant reductions in fatigue impact compared to the usual care control group.

### Specific aim 2

Our second aim is to determine whether 3-month use of MS INFoRm results in improvement in secondary outcomes of self- efficacy for managing MS fatigue, self-reported cognitive function, depression, and participation and autonomy. The measures we will use to evaluate these outcomes are the Multiple Sclerosis Self-Efficacy Scale (MSSES) [24], the Perceived Deficits Questionnaire (PDQ) [25], Center for Epidemiologic Studies Depression Scale (CES-D) [26], and Impact on Participation and Autonomy Questionnaire (IPA) [27] respectively. We hypothesize that the MS INFoRm group will show significant improvements in self-efficacy for managing MS fatigue, self-reported cognitive function, participation and autonomy, and significant reductions in depression, compared to the usual care control group.

### Specific aim 3

Our third and final aim is to determine whether any improvements in primary and secondary outcomes are maintained among the MS INFoRm users after 6-months. We hypothesize that the beneficial effects of MS INFoRm will be maintained at 6-month follow-up.

## Methods

### Study design, recruitment and location

This study will utilize a parallel group, two arm, superiority trial with a 1:1 allocation. Both participants and outcome assessors are blinded to group allocation. Participants will be recruited from across Canada via internet advertisements (MS Society of Canada Research Portal and MS group social media pages based in Canada). Participants will also be recruited from 3 MS clinics in Edmonton, Calgary, and Kingston located in academic hospitals (University of Alberta, University of Calgary, Queen’s University) by having MS clinic staff hand out study flyers. A last method of recruitment will be study flyers placed within the local Kingston, Ontario community. Our expected recruitment is 50 participants per site (Edmonton, Calgary, Kingston) and another 50 participants from internet advertisements. All recruitment methods will direct potential participants to contact the research team located at Queen’s University in Kingston, Ontario. All screening of participants, provision of website access and troubleshooting, and baseline and outcome assessment will be conducted by the research team at Queen’s University. Recruitment started in January 2018 and is anticipated to be completed by May 2019. If recruitment strategies are failing to generate necessary numbers of participants, backup sites for additional recruitment have been identified.

### Eligibility criteria

In order to be eligible for participation, an individual must: (1) have MS, (2) be between 18 and 65 years of age, (3) have access to a computer or other electronic device with internet access on which to review MS INFoRm, (4) have an average score between 2.0 and 5.4 across the 9 items of the Fatigue Severity Scale (FSS) [28], and (5) live in Canada. This last inclusion criteria is to minimize any confounding that may be introduced by differences in usual care across health care systems. Individuals will be excluded if they have any one of the following: (1) any major comorbid conditions that might influence fatigue management (lupus, rheumatoid arthritis, chronic obstructive lung disease, chronic fatigue syndrome), (2) report difficulty reading and comprehending English, (3) report upper extremity or visual impairments that cannot be accommodated adequately to enable computer access, (4) report depression based on a Patient Health Questionnaire-2 score [29] of 3 or more, (5) demonstrate evidence of cognitive impairment based on a Short Blessed Test [30] score or 12 or more.

### Group allocation

Group allocation will be based on a random permutated block design, using computerized random number generation. This design was selected because it ensures a 1:1 allocation ratio between the intervention group and the control group and because it will effectively reduce any risk of a systematic bias that may confound the treatment effect (i.e., people from the same MS clinic enrolling sequentially). The block size, which is fixed, will not be disclosed, to ensure concealment. One hundred people will be allocated to the MS INFoRm group and 100 to the usual care control group.

All eligible participants will be provided with a study identification number and password to access the MS INFoRm website. Using the block randomization process described above, each study ID has been assigned to one of the two groups by two of the study investigators. A password-protected file, and the MS INFoRm administrator website, which includes the group allocation for each Study ID, is only be accessible by two of the study investigators. Upon login, participants in the MS INFoRm intervention group will be directed to the intervention website whereas participants in the usual care control group will be directed to the usual care website. Participants will be provided with access to the website for 3-months from time of baseline assessment. Participants will access the website at their own volition, and not be directed on how often to use it as the nature of the intervention is self-directed, in order to replicate real-world use.

### MS INFoRm intervention website

A description of the sections, content, and features of the MS INFoRm intervention website is provided in Table 1.

**Table 1:** MS INFoRm intervention website content and features

Section	Primary Content and Features
1. Initial login page	- Presents introductory video providing user with orientation to website and overview of MS fatigue
2. Basics of fatigue	- Provides definition of fatigue - Provides facts about MS fatigue including prevalence, association with other disease-related factors, and how MS fatigue is different than regular fatigue - Provides description of primary and secondary fatigue - Includes section on tracking fatigue, including description of the benefits of tracking fatigue, how to track fatigue using a printable form, or on the “Rate your Fatigue” section (described below in section 3) of the website - Provides a link to the introductory video (enables users to review initial login page information)
3. Rate your fatigue	- Allows users to rate fatigue level at each visit (limits to one entry per day) from 0 (lowest fatigue) to 10 (highest fatigue) - Presents all ratings in a line graph that shows date and time, and rating
4. Track your goals	- After initial login page, users are prompted to set at least one fatigue management goal - Users are prompted to enter the following for each goal: (a) What do you want to do? (b) When do you want to start doing it? (c) How often do you want to do it? (d) How will you know that you have achieved this goal? (e) How confident are you that you can achieve this goal? - Users can update the answers to question (e) and are asked to rate the goal completion progress at each subsequent login - Users can have 3 goals active at a time, any goals that become achieved are put in a “Goals History” page - Users are prompted to select up to three fatigue management strategies to use to help them accomplish each goal. Strategies are described and can be selected from Sections 5 and 6 (described below) of the website
5. Influencing factors	Each of the following influencing factors, or contributors to secondary fatigue are provided on separate webpages
i. Poor sleep	- Describes the prevalence of poor sleep in persons with MS, causes of poor sleep (e.g. pain, stress, depression), definition and signs and symptoms of poor sleep, and common sleep disorders in persons with MS (insomnia, restless legs syndrome, and sleep apnea) - Describes when to talk to a healthcare professional regarding poor sleep - Describes 11 sleep hygiene strategies that can be used to improve sleep quality, and includes a link to a pdf sleep diary that can be used to track sleep and use of sleep hygiene strategies
ii. Medication side effects	- Describes how fatigue can be a side effect of medications - Describes use of medications for MS fatigue - Describes strategies for managing medication side effects and includes a link to a pdf medication side effects diary
iii. Deconditioning	- Defines physical activity, exercising, and deconditioning - Describes how getting enough physical activity can improve fatigue - Guides users about how to start an exercise program - Provides strategies for being more physically active, one of which includes working towards meeting the “Canadian Physical Activity Guidelines for People with MS” (description of these guidelines provided)
iv. Cognitive fatigue	- Provides a definition of cognitive fatigue, and contributing factors - Provides a description and examples of cognitive rehabilitation strategies that can help improve cognitive fatigue
v. Depression	- Provides a definition, and describes signs and symptoms, and prevalence of depression in MS - Describes when to get help for depression and includes a link to pdf version of the Patient Health Questionnaire-2. Users are encouraged to make an appointment with their health care provider if their score adds up to 3 or more - Describes how depression is treated in MS (pharmacological and non-pharmacological approaches) - Describes strategies for managing depression (e.g. participating in therapy, talking to healthcare provider about possibility of taking antidepressant medication)
6. General fatigue	- Describes principles of energy management and provides examples and strategies for how to bank and budget energy. A link to a pdf “Rest Scheduling Worksheet” is provided to help users with banking energy - Describes strategies for talking to other people about MS fatigue and asking for help, including five audio examples. Section also includes a link to a pdf handout which can be filled out and given to friends, family, or caregivers as a tool to teach others about MS fatigue and ask for help - Provides examples and strategies for modifying activities in order to use energy more wisely
7. Next steps	- Provides a summary of main points from website and things to remember - Provides quotes from people with MS who are working to manage their fatigue - Provides links to additional resources about MS fatigue (e.g. from MS Society of Canada, National MS Society, MS International Federation, etc.)

### Usual care website

The usual care website includes links to widely available brochures/ pdfs published by the MS Society of Canada entitled “MS Fatigue” [31] and “Living Well With MS: Managing Fatigue” [32]. These pdfs were chosen as they are resources to which clinicians, including those on the research team, often direct their MS patients. A brief description of the contents of each brochure/ pdf is provided on the website prior to presenting the link. The usual care website was designed to visually resemble the intervention website in order to facilitate blinding. We acknowledge, however, that some participants might be able to recognize that they have been allocated to the usual care control group if they have been previously exposed to the usual care resources.

### Primary outcome

A timeline of data collection of all measures is provided in Table 2. The primary outcome measure is the Modified Fatigue Impact Scale [23]. This 21-item scale assesses the impact of fatigue on daily functioning during the last 4 weeks. Each item asks about how fatigue has interfered with a particular life situation (e.g., “Because of my fatigue, I have been less motivated to participate in social activities.” “Because of my fatigue, I have been less able to complete tasks that require physical effort.”) Respondents indicate the impact of their fatigue on a scale of 0 (never) to 4 (almost always), resulting in a total score and three sub-scores (physical, cognitive and psychosocial). Higher scores indicate more severe impact of fatigue on daily life. The MFIS has been found to have good psychometric properties among people with MS [33].

**Table 2:** Time points and measures collected at study enrollment and three assessment time points for all participants

Measure	Time 0: Screening and study enrollment	Time 1: Baseline assessment^a^	Time 2: 3-month follow-up	Time 3: 6-month follow-up
Timepoint (months)	0	0^a^	3	9
Fatigue Severity Scale	X			
Patient Health Questionnaire – 2	X			
Short Blessed Test	X			
Demographic and disease-related information		X	X^b^	X^b^
Patient Determined Disease Steps Scale		X	X	X
Modified Fatigue Impact Scale		X	X	X
Multiple Sclerosis Self-Efficacy Scale		X	X	X
Perceived Deficits Questionnaire		X	X	X
Center for Epidemiologic Studies Depression Scale		X	X	X
Impact on Participation and Autonomy Questionnaire		X	X	X

^a^Within 1-week of study enrollment

^b^Modified to ask questions pertaining to changes to medications, rehabilitation services, employment status, and occurrence of MS relapse since last interview time point

### Secondary outcomes

Four secondary outcomes will be measured, including self-efficacy, perceived cognitive function, depression, and participation and autonomy. Self-efficacy will be measured using the Multiple Sclerosis Self-Efficacy Scale (MSSES) [24], which consists of 14 items. Respondents rate the degree to which they believe they can overcome challenges using a 6-point Likert scale, ranging from “strongly disagree” to “strongly agree”. A high score indicates high self-efficacy. Validity, reliability and sensitivity to change of the MSSES has been established [24].

The Perceived Deficits Questionnaire (PDQ) will be used to measure perceived cognitive function [25]. The full-length PDQ consists of 20 items assessing the cognitive functions most commonly affected in MS: attention, retrospective memory, prospective memory, and planning and organization. Respondents rate problems in these areas according to their frequency from 0 (never) to 4 (almost always) in the past 4 weeks, with higher scores indicating worse perceived cognitive functioning. The PDQ was developed specifically for persons with MS [25], and is associated with objective cognitive performance [34].

The Center for Epidemiologic Studies Depression Scale (CES-D) will be used to measure depression [26]. The CES-D has been found to be a valid and reliable scale to assess depressive symptomatology in MS [35]. This measure consists of 20 items describing depression-related symptoms that are rated according to frequency in the last week from 0 (rarely or none of the time) to 3 (all of the time). A cut-off score of 16 or higher is suggestive of clinically significant depressive symptoms [26].

The Impact on Participation and Autonomy Questionnaire (IPA) will be used to measure participation and autonomy [27, 36]. The IPA provides a measure of limitations in participation and autonomy and is a commonly used measure for evaluating rehabilitation outcomes in individuals with chronic disease, including MS [37, 38]. The tool includes 39 questions across 5 domains: autonomy indoors, autonomy outdoors, family role, social life and relationships, and work and education. Participants rate each item on scale from 0 (very good) to 4 (very poor). The scoring captures how likely respondents feel they will be able to participate in a described activity or how their disability impacts their ability to participate. Higher scores indicate greater restrictions. The IPA has been shown to have strong psychometric properties, including reliability, validity and responsiveness to intervention [36, 37, 39].

### Sample size

We seek to recruit a total of 200 individuals. The total number of individuals to be recruited is based on calculations of sample size and power for a balanced independent-groups mixed effect model with three time points for our primary outcome (i.e., fatigue impact at baseline, 3 months, 6-months post use). The effect sizes, variances and covariance estimates were obtained from our pilot results [21]. Calculations take into account potential attrition over time, which could be as high as 34% based on our pilot study. This high attrition rate, however, was likely due to limited persistence of research staff for follow-up assessments, which we will attempt to remediate in the proposed project.

### Data collection and management

Screening, baseline, 3-month, and 6-month interviews will be conducted by research assistants, each provided with same training, and blinded to group allocation. Reminders will be given to participants 2–3 days prior to each interview. Four attempts, using different methods of contact, will be made for participants we have been unable to reach for 3-month or 6-month follow-up interviews. For individuals who withdraw from the study, the reasons for withdrawal will be documented, if discoverable.

At screening, each individual will be assigned a Study ID that will be used for all subsequent data collection and analysis in order to maintain anonymity. A list linking Study ID to personal identifying information (name, age, contact information) will be stored in a password-protected file to which only two of the study investigators and the research assistants will have access to. Hard copies of interview data will be kept in a locked filing cabinet at the main site (Queen’s University). As data are collected during the study, the research assistants will code and enter the data into a database created in IBM SPSS Statistics Version 24. All data files will be stored on an encrypted drive on the Queen’s University server to which only two of the study investigators and research assistants will have access. Data back-ups will occur daily using an encrypted external hard drive.

### Website usage data

User analytic data will be collected from the website (from both groups) in order to track the number of times the site is accessed, length of each visit, specific pages visited, time spent on each page, which worksheets and links were accessed and how often, and to capture all fatigue ratings and goal data entered. These data will be collected to identify delivery fidelity (did participants access all components of the website) and for descriptive analysis of usage patterns and impact of use on outcomes.

### Statistical analysis

All data will be analyzed in IBM SPSS Statistics Version 24. Scores on all outcome measures will be calculated according to manual instructions for each measure. Unusually high or low values will be checked against the raw data to confirm accuracy. Descriptive statistics will be calculated for each variable for both the intervention and usual care control group. Score distributions will be checked to ensure that distributional assumptions are met for the hypothesis testing step of the analysis.

The MS INFoRm and usual care groups will be compared on baseline measures and key demographic and disease-related variables that may influence study outcomes (e.g. age, disease duration, education level) to test equivalence of groups. Independent samples t-tests will be used for continuous variables with a normal distribution, Mann Whitney U tests for non-normally distributed data, and chi-square tests for categorical variables. If differences are found, these variables will be used as covariates during hypothesis testing to ensure that group differences at baseline are not responsible for differences in outcomes.

For individuals lost to follow-up, their data will be used in the statistical analysis in the effectiveness analysis as per an intention to treat approach [40]. These individuals will remain in the same group as randomized. The advantage of this approach is that the estimated effect of the intervention will reflect the effect that would actually be achieved in the real world (i.e., effectiveness analysis) when people have differential motivation to use MS INFoRm. Missing data will be imputed using the strategy of last observation carried forward, as this is the most conservative [41].

Hypothesis 1 states that the MS INFoRm group will show significant reductions in fatigue impact compared to the usual care control group. In order to test hypothesis 1, changes between baseline and 3-month scores on the MFIS will be compared between the MS INFoRm group and the usual care control group using an independent t-test. If the testing for group equivalence indicates that there are baseline differences between the groups at baseline, an analysis of variance with covariates will be used to test hypothesis 1. A *p* < 0.05 will be used as the significance value. In order to test efficacy, or whether MS INFoRm works when delivered as intended, data from only those individuals who have complete baseline and 3-month data and have accessed all of the website content at least once will be used in the analysis. In order to test effectiveness, participants who have missing 3-month scores will be assigned a change score of zero.

Hypothesis 2 states that the MS INFoRm group will show significant improvements in self-efficacy for managing MS fatigue, self-reported cognitive function, participation and autonomy, and significant reductions in depression, compared to the usual care control group. The analysis will use the same approach as hypothesis 1, using zero-value change scores for individuals lost to follow-up. A Bonferroni adjustment will be applied for multiple testing, and therefore the significance value will be set at *p* < 0.0125 (0.05/4 tests).

Hypothesis 3 states that the beneficial effects of MS INFoRm will be maintained at 6-month follow-up. To address this hypothesis, a mixed-effect ANOVA will be used with an unstructured variance-covariance structure, a random intercept and terms for both a linear and quadratic trend. A quadratic trend would be characterized by a sharp improvement immediately post-intervention (3-months), followed by a gradual stabilization of improvement. The five measures (MFIS, MSSES, PDQ, CES-D, and IPA) will be modelled simultaneously and individually. When modelled simultaneously, a significance value of *p* < 0.05 will be used. When modelled individually, a Bonferroni adjustment will be applied for multiple testing, and therefore the significance value will be set at *p* < 0.01 (0.05/5 tests).

### Research ethics approval

This trial has already received research ethics approval from the Queen’s University Health Sciences & Affiliated Teaching Hospitals Research Ethics Board (HSREB), the University of Alberta Health Research Ethics Board- Health Panel, and the University of Calgary Conjoint Health Research Ethics Board (CHREB). Any protocol modifications (e.g. changes in eligibility criteria, participant handouts, study personnel) will be submitted as amendments to all ethics committees. Written informed consent to participate will be obtained by the research assistants prior to baseline data collection for individuals screened and deemed eligible for the study. These consent forms will be stored in a locked filing cabinet at the main site (Queen’s University), separately from other data collected for the study.

As there are no major harms or risks associated with this study, no data monitoring committee will be formed. Furthermore, no provision of ancillary and post-trial care, interim analysis, or formal auditing is planned. However, monthly teleconferences will be scheduled with all study investigators in order to provide updates on recruitment, data collection, and analysis, and any difficulties encountered throughout the trial (e.g. with recruitment, withdrawals).

### Dissemination plan

Academic dissemination strategies will be pursued such as local, national, and international conference presentations and peer-reviewed journal articles. Authorship eligibility will be determined based on criteria of the International Committee of Medical Journal Editors [42]. We will also disseminate findings via social media and sharing with appropriate stakeholders. Should the results warrant (i.e. hypotheses are supported), we will work with our website developers and business innovators to find ways to maintain the website and make it broadly available to people with MS through MS clinics, MS support groups and other networks for adults living with MS. The trial protocol is listed in the ClinicalTrials.gov database (identifier: NCT03362541). Final trial data will only be accessible to the study investigators. However, individual data requests can be submitted by contacting the principal investigator directly.

## Discussion

If this trial shows that the MS INFoRm website is effective, it could lead to major improvements in access to fatigue management information for people with MS in Canada and beyond. Fatigue is one of the most common and disabling symptoms experienced by people with MS. While this resource will not cure MS or MS fatigue, it is important to have easily accessible and individualized options for fatigue management that people with MS can access and return to whenever they need the information. If effective, the MS INFoRm website could be made widely available to people living with MS and its format could be used as a model to create similar resources for the management of other MS symptoms.

With respect to limitations, difficulties could be encountered with respect to recruitment of our anticipated target sample size of 200 participants. As such, we have an additional back-up site if needed. Further hindering our recruitment efforts could be the strict inclusion/ exclusion criteria for this study, especially with respect to fatigue severity. We note, however, that MS INFoRm is not intended for everyone, but is specifically targeted to those reporting mild to moderate levels of fatigue. Other potential limitations include that some participants in the usual care control group may become un-blinded upon seeing the usual care webpage despite our efforts to make both the intervention and control pages look similar. It is possible that participants may share login information with others, which we cannot control or track. There is possibility of technical problems as new versions of browsers or devices become available. To minimize this risk, we will conduct regular checks of the website functionality and work closely with the web developer to make decisions about site changes if they become necessary because of technology updates. Finally, due to lack of supervision when using the website, users may get distracted and leave the website idle, which may bias any descriptive analysis of user analytic data. As a potential safeguard against this, users will be logged out and forced to log back in again after 30 min of non-use. Despite such limitations, using a web-based platform will make MS INFoRm a widely accessible tool for persons with MS trying to manage debilitating fatigue.

## Data Availability

Final trial data will only be accessible to the study investigators. However, individual data requests can be submitted by contacting the principal investigator (MF) directly.

## Abbreviations

CES-D: Center for Epidemiologic Studies Depression Scale
IPA: Impact on Participation and Autonomy Questionnaire
MFIS: Modified Fatigue Impact Scale
MS INFoRm: Multiple Sclerosis: An Interactive Fatigue Management Resource
MS: multiple sclerosis
MSSES: Multiple Sclerosis Self-Efficacy Scale
PDQ: Perceived Deficits Questionnaire

## References

[1] Multiple Sclerosis Clinical Practice Guidelines Council. Fatigue and multiple sclerosis: evidence-based management strategies for fatigue in multiple sclerosis. Washington, DC: Paralyzed Veterans Association; 1998.

[2] Weiland TJ, Jelinek GA, Marck CH, Hadgkiss EJ, Van Der Meer DM, Pereira NG (2015). Clinically significant fatigue: prevalence and associated factors in an international sample of adults with multiple sclerosis recruited via the internet. PLoS One.

[3] Coyne KS, Boscoe AN, Currie BM, Landrian AS, Wandstrat TL (2015). Understanding drivers of employment changes in a multiple sclerosis population. Int J MS Care.

[4] Benedict RH, Wahlig E, Bakshi R, Fishman I, Munschauer F, Zivadinov R (2005). Predicting quality of life in multiple sclerosis: accounting for physical disability, fatigue, cognition, mood disorder, personality, and behavior change. J Neurol Sci.

[5] Morrison JD, Stuifbergen AK (2016). Predictors of fatigue impact in persons with long-standing multiple sclerosis. J Neurosci Nurs.

[6] Finlayson M, Johansson S, Kos D, Finlayson M (2013). Fatigue. Multiple sclerosis rehabilitation: from impairment to participation.

[7] McLaughlin J, Zeeberg I (1993). Self-care and multiple sclerosis: a view from two cultures. Soc Sci Med.

[8] Coote S, Garrett M, Hogan N, Larkin A, Saunders J (2009). Getting the balance right: a randomised controlled trial of physiotherapy and exercise interventions for ambulatory people with multiple sclerosis. BMC Neurol.

[9] Marrie RA, Reingold S, Cohen J, Stuve O, Trojano M, Sorensen PS (2015). The incidence and prevalence of psychiatric disorders in multiple sclerosis: a systematic review. Mult Scler J.

[10] Johansson S, Ytterberg C, Gottberg K, Holmqvist LW, von Koch L (2009). Use of health services in people with multiple sclerosis with and without fatigue. Mult Scler.

[11] Kos D, Kerckhofs E, Nagels G, D’hooghe MB, Ilsbroukx S (2008). Origin of fatigue in multiple sclerosis: review of the literature. Neurorehabil Neural Repair.

[12] Asano M, Berg E, Johnson K, Turpin M, Finlayson ML (2014). A scoping review of rehabilitation interventions that reduce fatigue among adults with multiple sclerosis. Disabil Rehabil.

[13] Moss-Morris R, McCrone P, Yardley L, van Kessel K, Wills G, Dennison L (2012). A pilot randomised controlled trial of an internet-based cognitive behavioural therapy self-management programme (MS Invigor8) for multiple sclerosis fatigue. Behav Res Ther.

[14] Asano M, Finlayson ML (2014). Meta-analysis of three different types of fatigue management interventions for people with multiple sclerosis: exercise, education, and medication. Mult Scler Int.

[15] Thomas PW, Thomas S, Kersten P, Jones R, Slingsby V, Nock A (2014). One year follow-up of a pragmatic multi-centre randomised controlled trial of a group-based fatigue management programme (FACETS) for people with multiple sclerosis. BMC Neurol.

[16] Mathiowetz VG, Finlayson ML, Matuska KM, Chen HY, Luo P (2005). Randomized controlled trial of an energy conservation course for persons with multiple sclerosis. Mult Scler.

[17] Ghahari S, Packer T, Passmore AE (2010). Effectiveness of an online fatigue self-management programme for people with chronic neurological conditions: a randomized controlled trial. Clin Rehabil.

[18] Merriam SB. Andragogy and self-directed learning: pillars of adult learning theory. New Dir Adult Contin Educ. 2001;(89):3–14. 10.1002/ace.3.

[19] Bodenheimer T, Lorig K, Holman H, Grumbach K (2002). Patient self-management of chronic disease in primary care. JAMA.

[20] Lorig KR, Holman HR (2003). Self-management education: history, definition, outcomes, and mechanisms. Ann Behav Med.

[21] Akbar N, Turpin K, Petrin J, Smyth P, Finlayson M. A pilot mixed-methods evaluation of MS INFoRm: a self-directed fatigue management resource for individuals with multiple sclerosis. Int J Rehabil Res. 2018;41(2):114-21. 10.1097/MRR.0000000000000271. 10.1097/MRR.0000000000000271 29324506

[22] Petrin J, Akbar N, Turpin K, Smyth P, Finlayson M. The experience of persons with multiple sclerosis using MS INFoRm: an interactive fatigue management resource. Qual Health Res. 2018. 10.1177/1049732317753584. 10.1177/1049732317753584 29411682

[23] Ritvo PG, Fischer JS, Miller DM, Andrews H, Paty DW, LaRocca NG (1997). Multiple sclerosis quality of life inventory: a user’s manual.

[24] Rigby S, Domenech C, Thornton E, Tedman S, Young C. Development and validation of a self-efficacy measure for people with multiple sclerosis: the multiple sclerosis self-efficacy scale. Mult Scler J. 2003;9:73–81. 10.1191/1352458503ms870oa. 10.1191/1352458503ms870oa 12617272

[25] Sullivan MJ, Edgely K, Dehoux E (1990). A survey of multiple sclerosis. Part 1: perceived cognitive problems and compensatory strategy use. Can J Rehabil.

[26] Radloff LS (1977). The CES-D scale: a self-report depression scale for research in the general population. Appl Psychol Meas.

[27] Cardol M, Haan RJ d, van den Bos GA, Jong BA d, de Groot IJ (1999). The development of a handicap assessment questionnaire: the impact on participation and autonomy (IPA). Clin Rehabil.

[28] Krupp LB, LaRocca NG, Muir-Nash J, Steinberg AD (1989). The fatigue severity scale. Application to patients with multiple sclerosis and systemic lupus erythematosus. Arch Neurol.

[29] Kroenke K, Spitzer RL, Williams JBW (2003). The patient health Questionnaire-2 validity of a two-item depression screener. Med Care.

[30] Katzman R, Brown T, Fuld P, Peck A, Schechter R, Schimmel H (1983). Validation of a short orientation-memory-concentration test of cognitive impairment. Am J Psychiatry.

[31] Multiple Sc lerosis Society of Canada. MS fatigue. https://mssociety.ca/en/pdf/fatigue-info-sheet.pdf. Accessed 2 Mar 2018.

[32] Krupp LB for the Multiple Sclerosis Society of Canada. Living well with MS: managing fatigue. https://mssociety.ca/en/pdf/livingWell.pdf. Accessed 2 Mar 2018.

[33] Learmonth YC, Dlugonski D, Pilutti LA, Sandroff BM, Klaren R, Motl RW (2013). Psychometric properties of the fatigue severity scale and the modified fatigue impact scale. J Neurol Sci.

[34] Marrie R, Chelune G, Miller D, Cohen J (2005). Subjective cognitive complaints relate to mild impairment of cognition in multiple sclerosis. Mult Scler.

[35] Verdier-Taillefer MH, Gourlet V, Fuhrer R, Alperovitch A (2001). Psychometric properties of the Center for Epidemiologic Studies-Depression scale in multiple sclerosis. Neuroepidemiology.

[36] Cardol M, De Haan RJ, De Jong BA, Van den Bos GAM, De Groot IJM (2001). Psychometric properties of the impact on participation and autonomy questionnaire. Arch Phys Med Rehabil.

[37] Kersten P, Cardol M, George S, Ward C, Sibley A, White B (2007). Validity of the impact on participation and autonomy questionnaire: a comparison between two countries. Disabil Rehabil.

[38] Sibley A, Kersten P, Ward C, B W, Mehta R, Alexopoulos GS (2006). Measuring autonomy in disabled people: validation of a new scale in a UK population. Clin Rehabil.

[39] Cardol M, Beelen A, Van den Bos GA, De Jong BA, De Groot IJ, De Haan RJ (2002). Responsiveness of the impact on participation and autonomy questionnaire. Arch Phys Med Rehabil.

[40] Lachin JM (2000). Statistical considerations in the intent-to-treat principle. Control Clin Trials.

[41] Little R, Yau L (1996). Intent-to-treat analysis for longitudinal studies with drop-outs. Biometrics.

[42] International Committee of Medical Journal Editors. Defining the role of authors and contributors. http://www.icmje.org/recommendations/browse/roles-and-responsibilities/defining-the-role-of-authors-and-contributors.html. Accessed 2 Mar 2018.

